# Gonadotropin-releasing hormone agonist downregulation combined with hormone replacement therapy improves the reproductive outcome in frozen–thawed embryo transfer cycles for patients of advanced reproductive age with idiopathic recurrent implantation failure

**DOI:** 10.1186/s12958-022-00897-3

**Published:** 2022-02-03

**Authors:** Dan Pan, Jie Yang, Ni Zhang, Lei Wang, Na Li, Juanzi Shi, Hanying Zhou

**Affiliations:** 1 The Assisted Reproduction Center, Northwest Women’s and Children’s Hospital, No. 73 Hou zai Gate, Xin cheng District, Xi’an City, 710004 Shaanxi Province China; 2 Department of Basic Medicine, Xi’an Medical University, Xin-Wang Street #1, Xi’an, 710021 Shaanxi China

**Keywords:** Frozen–thawed embryo transfer, Recurrent implantation failure, Gonadotropin-releasing hormone agonist, Hormone replacement therapy

## Abstract

**Background:**

To determine whether gonadotropin-releasing hormone (GnRH) agonist downregulation combined with hormone replacement therapy (HRT) can improve the reproductive outcomes in frozen–thawed embryo transfer cycles for older patients (aged 36–43 years) with idiopathic recurrent implantation failure (RIF).

**Methods:**

This retrospective cohort study involved 549 older patients undergoing their third cleavage-stage embryo or blastocyst transfer over a 5-year period (January 2015–December 2020) at Northwest Women’s and Children’s Hospital after in vitro fertilization/intracytoplasmic sperm injection cycles. Patients with known endometriosis or adenomyosis were excluded from the study. The patients were divided into three groups according to the endometrial preparation protocol: the natural cycle (NC) group (*n* = 65), the HRT group (*n* = 194), and the GnRH agonist downregulation combined with HRT cycle (GnRH agonist–HRT) group (*n* = 290). The primary outcome was the live birth rate, and the secondary outcomes were the clinical pregnancy, miscarriage, and ongoing pregnancy rates.

**Results:**

The live birth rate in the GnRH agonist–HRT group (36.55%) was higher than that in the HRT group (22.16%) and NC group (16.92%) (*P* < 0.0001). Similarly, a logistic regression model adjusting for potential confounders showed that the live birth rate was higher in the GnRH agonist–HRT group than in the HRT group (odds ratio, 0.594; 95% confidence interval, 0.381–0.926; *P* = 0.021) and NC group (odds ratio, 0.380; 95% confidence interval, 0.181–0.796; *P* = 0.010).

**Conclusions:**

The GnRH agonist–HRT protocol improves the live birth rate in frozen–thawed embryo transfer cycles for patients of advanced reproductive age with RIF. We hypothesize that the GnRH agonist–HRT protocol enhances implantation-related factors and promotes optimal endometrial receptivity, leading to an improved live birth rate. These findings are also useful for further investigating the underlying mechanism of the GnRH agonist–HRT protocol in improving the reproductive outcomes for patients of advanced reproductive age with RIF.

**Trial registration:**

This research protocol was approved by the hospital institutional ethics committee (No. 2021002).

## Background

Despite technical advances in in vitro fertilization (IVF), the management of idiopathic recurrent implantation failure (RIF)—the absence of implantation after repeated embryo transfers—poses a major clinical challenge in assisted reproductive technology (ART). No clear international consensus on the definition of RIF or optimal therapeutic approaches has yet been established [1, 2]. The diagnosis of more than two transplantation failures, as a common pattern, is recommended by 23 to 45% of ART specialists [3].

The endometrium is the destination of embryo implantation, and successful implantation requires the embryo to interact with a receptive endometrial lining. The two interact and influence each other while the embryo enters the uterine cavity [4]. Therefore, both adequate endometrial receptivity and high embryonic quality are equally important for implantation. Prospective cohort studies have indicated that frozen–thawed embryo transfer (FET) might improve the reproductive outcomes in women with RIF [5]. In FET cycles, the embryos may be transferred into a more synchronous uterine environment, which can avoid the disturbed endometrial development caused by external ovarian stimulation.

Which endometrial preparation protocol is more effective to promote implantation remains controversial, especially in women of advanced reproductive age (≥36 years of age) with RIF. A previous retrospective self-control study showed that a gonadotropin-releasing hormone agonist combined with hormone replacement therapy (GnRH agonist–HRT) protocol can increase the pregnancy success rate in FET cycles of patients who have experienced RIF following IVF treatment [6]. Other studies have shown that a depot GnRH agonist protocol might protect the expression of endometrial receptivity markers and thereby improve endometrial receptivity [7, 8]. Nevertheless, these findings were questioned in a further retrospective cohort study showing that a GnRH agonist–HRT protocol does not improve the live birth rate following ART for patients with RIF. However, patients of all ages were included in that study [9].

Moreover, increasing maternal age as another risk factor for embryo–endometrium asynchrony makes successful embryo implantation even more complicated [10, 11]. As one of the main indicators of RIF, the effects of female age have been discussed widely [12]. However, few studies have focused on reproductive outcomes in older populations with RIF. We hypothesized that before FET, older patients with idiopathic RIF might benefit from single-agent GnRH agonist suppression compared with those without such pretreatment.

## Methods

### Study design and patients

**Fig. 1:**
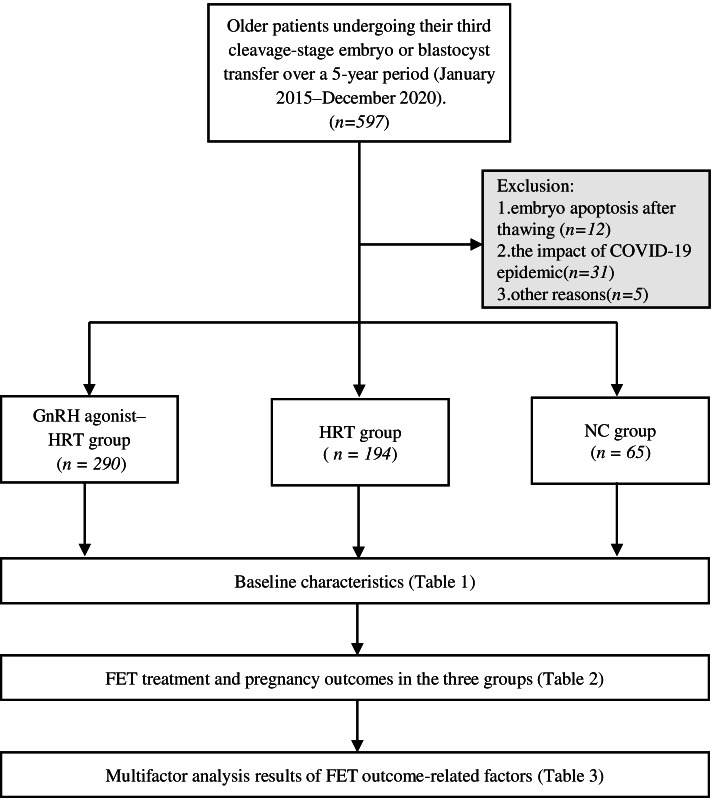
Data collection and analysis method

This study included older patients (36–43 years of age) undergoing FET cycles at Northwest Women’s and Children’s Hospital after IVF/intracytoplasmic sperm injection (ICSI) cycles from January 2015 to December 2020. This study only included patients undergoing the third or more embryo transfer after autologous IVF or ICSI (*n* = 597). Women who underwent embryo transfer after receiving donor oocytes; women who were diagnosed with endometriosis, adenomyosis, or both; and women whose endometrial thickness did not reach 7 mm on the day of transplantation were excluded. In addition, 48 women were excluded mainly because of embryo apoptosis after thawing, the impact of the COVID-19 pandemic, or other reasons (Fig. 1). The research protocol was approved by the hospital’s institutional ethics committee (No. 2021002).

### Endometrial preparation before FET

In patients who underwent FET during natural cycles (NCs) (including modified cycles; NC group), we monitored when the leading follicles reached a mean diameter of > 17 mm by transvaginal ultrasonography. Human chorionic gonadotropin (10,000 IU) was injected to trigger ovulation when the serum luteinizing hormone level was < 20 IU/L; otherwise, transvaginal ultrasonography was performed every day until ovulation. FET was performed 3 days (cleavage-stage embryos) or 5 days (blastocysts) after ovulation.

In cycles with HRT (HRT group), starting at cycle day 5, estradiol valerate (Progynova; Bayer Schering Pharma AG, Berlin, Germany) was administered orally at 4 to 6 mg daily. Approximately 10 to 12 days later, 60 mg/day of natural progesterone in oil (Xianju, Zhejiang, China) was injected intramuscularly to prepare the endometrium as soon as the endometrial thickness reached 7 mm and the serum progesterone level was < 1.5 ng/mL. FET was then performed after 4 days (cleavage-stage embryos) or 6 days (blastocysts) of progesterone therapy.

In cycles with GnRH agonist downregulation combined with HRT (GnRH agonist–HRT group), patients received a single injection of 3.75 mg long-acting triptorelin acetate on day 2 of the cycle after an ultrasound scan confirmed ovarian quiescence and the presence of a thin endometrium (< 5 mm). After 28 to 30 days, estrogen stimulation was used as in the HRT cycles.

### Embryo and blastocyst cryopreservation

All embryos were preserved by vitrification and thawed when transferred. The cleavage-stage embryos were warmed in the afternoon 1 day before embryo transfer with a post-warm-culture time of 18 to 20 h. The blastocysts were warmed on the day of embryo transfer with a post-thaw culture time of 2 h.

### Luteal support

Luteal support schemes for the GnRH agonist–HRT group and HRT group included intramuscular injection of 60 mg/day of natural progesterone in oil and oral intake of 20 mg/day progesterone tablets (Dydrogesterone; Abbott Biologicals B.V., The Netherlands) [13, 14]. The dosage of progesterone decreased to 40 mg/day and the progesterone tablet dosage was the same as before in the NC/modified NC group. After embryo transfer, all patients received luteal support until day 14 when the plasma β-human chorionic gonadotropin concentration was determined. If this was positive, hormone administration was continued until 10 weeks of pregnancy.

### Outcome parameters

The primary outcome parameter was the live birth rate. The delivery of a viable infant after the 22nd gestational week was considered a live birth. The secondary outcomes were the clinical pregnancy rate (defined as the rate of an intrauterine gestational sac with fetal heart activity on ultrasound), the ongoing pregnancy rate (defined as a viable pregnancy past 12 weeks of gestation), and the miscarriage rate (the loss of a clinical pregnancy before the 22nd gestational week per clinical pregnancy cycle) [15].

### Statistical analysis

Analysis of variance was used for continuous variables (mean ± standard deviation) and the χ^2^ test was used for categorical variables to determine whether the distributions of covariates were statistically significant across treatment protocols. Multivariable logistic regression was used to identify the independent effect of the treatment protocol on the live birth rate and miscarriage rate in patients with RIF, adjusting for patient age, body mass index, embryo transfer cycle number, interval between fresh and frozen transfer, basal follicle-stimulating hormone level, infertility duration in years, infertility type, endometrial thickness on the day of FET, number of embryos transferred, and embryo transfer type. The results are shown as the odds ratio (OR) and 95% confidence interval (CI). A *P* value of < 0.05 was considered statistically significant. Data processing and statistical analysis were conducted using IBM SPSS Statistics v. 19.0 (IBM Corp., Armonk, NY, USA).

## Results

In total, 549 women were enrolled in the study. The demographic and clinical characteristics of the patients are listed in Table 1. There were no significant differences in their age at FET, body mass index, embryo transfer cycle number, interval between fresh and frozen transfer, basal follicle-stimulating hormone level, duration of infertility, type of infertility, numbers of embryos transferred, or proportion of transferred blastocysts among the three groups. The level of progesterone on the administration day was significantly lower in the GnRH agonist–HRT group than in the HRT group (*P* = 0.000). In addition, fewer embryos were formed by ICSI insemination in the NC group than in the other two groups (*P* = 0.028). Furthermore, the endometrial thickness on the day of FET was significantly different among the three groups (*P* = 0.000).

**Table 1:** Baseline characteristics between different endometrial preparation groups

Characteristic	GnRH agonist-HRT	HRT	NC	*P* value
	(*n* = 290)	(*n* = 194)	(*n* = 65)	
female age(y)	38.7 ± 2.19	39.14 ± 2.2	38.74 ± 1.96	0.083
BMI (kg/m2)	22.37 ± 2.74	22.66 ± 2.77	22.13 ± 2.48	0.313
ET cycle number	3.43 ± 0.75	3.42 ± 0.71	3.52 ± 0.9	0.609
Interval between fresh and frozen transfer(m)	6.41 ± 5.92	6.11 ± 6.57	5.75 ± 4.65	0.688
bFSH (IU/L)	7.95 ± 2.97	8.28 ± 2.96	8.07 ± 3.17	0.509
Infertility duration in years (y)	5.48 ± 4.10	5.27 ± 4.28	5.78 ± 3.98	0.670
Infertility type (n, %)				0.272
Primary infertility	75 (25.86)	46 (23.71)	22 (33.85)	
Secondary infertility	215 (74.14)	148 (76.29)	43 (66.15)	
Fertilization type (n, %)				0.028 *
IVF	208 (71.70)	135 (69.60)	49 (75.40)	
ICSI	78 (26.90)	56 (28.90)	11 (16.90)	
IVF + ICSI	4 (1.40)	3 (1.50)	5 (7.70)	
Progesterone on the administration day (ng/ml)	0.38 ± 0.29	0.54 ± 0.47		0.000 *
Endometrial thickness on the day of transfer (mm)	10.34 ± 1.68	9.79 ± 1.54	10.78 ± 2.25	0.000 *
No. of transferred embryos (n, %)				0.055
1	84 (28.97)	70 (36.08)	29 (44.62)	
2	193 (66.55)	116 (59.79)	31 (47.69)	
3	13 (4.48)	8 (4.12)	5 (7.69)	
Type of embryo transferred(n, %)				0.074
Cleavage stage embryo	130 (44.83)	107 (55.15)	34 (52.31)	
Blastocyst	160 (55.17)	87 (44.85)	31 (47.69)	

Values are means±SD or number (percentage) of patients. *P < 0.05 for t test or chi-squared test;

Abbreviations: *BMI* Body mass index; *bFSH* basal follicle-stimulating hormone; *IVF* In vitro fertilization; *ICSI* Intracytoplasmic sperm injection

The clinical pregnancy, ongoing pregnancy, and live birth rates were significantly higher in the GnRH agonist–HRT group than in the other two groups. However, the miscarriage rates did not differ significantly among the three groups (Table 2).

**Table 2:** FET treatment and pregnancy outcomes in the three groups

Pregnancy outcomes	GnRH agonist-HRT	HRT	NC	*P* value
Clinical pregnancy rate	48.97 (142/290)	35.05 (68/194)	27.69 (18/65)	0.001 *
Ongoing pregnancy rate	37.59 (109/290)	22.68 (44/194)	18.46 (12/65)	0.000 *
Miscarriage rate	21.83 (31/142)	36.76 (25/68)	33.33 (6/18)	0.062
Live birth rate	36.55 (106/290)	22.16 (43/194)	16.92 (11/65)	0.000 *

Values are percentage (no./no.) of patients. *P < 0.05 for chi-squared test

In the adjusted model, the GnRH agonist–HRT treatment protocol resulted in better clinical pregnancy, ongoing pregnancy, and live birth rates than did the other two protocols. In addition, the patient age, number of embryos transferred, and embryo transfer type were still related to the live birth rate (Table 3).

**Table 3:** Multivariate logistic regression analysis of factors related to pregnancy outcomes of FET

Clinical indicators	Clinical pregnancy rate		Ongoing pregnancy rate	
Value	OR value (95% CI)	*P* value	OR value (95% CI)	*P* value
Female age(y)	0.868 (0.796-0.947)	0.001*	0.813 (0.738-0.896)	0.000*
Fertilization type				
IVF	Ref.		Ref.	
ICSI	0.815 (0.536-1.241)	0.340	0.867 (0.547-1.376)	0.546
IVF + ICSI	0.579 (0.159-2.104)	0.406	1.072 (0.286-4.022)	0.918
Endometrial thickness on the day of transfer (mm)	1.059 (0.952-1.179)	0.291	1.026 (0.913-1.152)	0.669
No. of transferred embryos				
1	Ref.		Ref.	
2	2.495 (1.642-3.791)	0.000*	2.974 (1.853-4.773)	0.000*
3	1.92 (0.763-4.832)	0.166	1.754 (0.579-5.306)	0.320
Type of embryo transferred				
Cleavage stage embryo	Ref.		Ref.	
Blastocyst	2.047 (1.388-3.02)	0.000*	2.263 (1.479-3.461)	0.000*
Endometrial Preparation Protocols				
GnRH agonist-HRT	Ref.		Ref.	
HRT	0.664 (0.446-0.987)	0.043*	0.573 (0.369-0.891)	0.013*
NC	0.439 (0.235-0.822)	0.010*	0.414 (0.202-0.846)	0.016*
Clinical indicators	Live birth rate			
Value	OR value(95% CI)	*P* value		
Female age(y)	0.819 (0.742-0.903)	0.000 *		
Fertilization type				
IVF	Ref.			
ICSI	0.884 (0.555-1.409)	0.605		
IVF + ICSI	1.119 (0.298-4.204)	0.868		
Endometrial thickness on the day of transfer (mm)	1.057 (0.94-1.188)	0.352		
No. of transferred embryos				
1	Ref.			
2	3.032 (1.877-4.897)	0.000 *		
3	1.89 (0.622-5.742)	0.261		
Type of embryo transferred				
Cleavage stage embryo	Ref.			
Blastocyst	2.28 (1.485-3.5)	0.000 *		
Endometrial Preparation Protocols				
GnRH agonist-HRT	Ref.			
HRT	0.594 (0.381-0.926)	0.021 *		
NC	0.38 (0.181-0.796)	0.010 *		

*P < 0.05 for logistic regression

Abbreviations: *OR* Odds ratio; *CI 95%* Confidence interval; *IVF* In vitro fertilization; *ICSI* Intracytoplasmic sperm injection

## Discussion

The lack of a clearly defined treatment for women with RIF is well known. There is insufficient evidence that the use of a depot GnRH agonist combined with an HRT cycle is better or worse than the NC or HRT protocol for reproductive outcomes in FET cycles, especially for patients with RIF. Pretreatment with a GnRH agonist before HRT in FET cycles is considered ineffective for patients of advanced reproductive age, but this does not include patients with RIF [16]. A recent study also failed to demonstrate any significant effect of a depot GnRH agonist–HRT protocol on the live birth/ongoing pregnancy rate for patients with RIF [9]. However, that study only included younger patients (< 40 years of age); thus, the effect of a depot GnRH agonist–HRT protocol on older patients (36–43 years of age) needs to be further evaluated.

This large-scale retrospective cohort study evaluated the reproductive outcomes in the NC, HRT cycle, and GnRH agonist–HRT cycle of patients of advanced reproductive age with RIF when referred for FET. The live birth rate was higher with the GnRH agonist–HRT treatment protocol than with the NC and HRT protocols, whereas the miscarriage rate was not significantly different among the three protocols. In addition, we used a single-dose drug intramuscular injection of 3.75 mg depot leuprolide acetate. This is consistent with a previous retrospective cohort analysis that suggested that a GnRH agonist–HRT protocol with 28 days of a full-dose depot GnRH agonist, giving prolonged downregulation before ovarian stimulation, resulted in relatively high pregnancy success rates in patients with idiopathic RIF undergoing IVF [6].

GnRH agonists might regulate the expression of endometrial adhesive molecules, implantation markers, and genetic alterations. HOXA10 and MEIS, well-known markers, can improve uterine development and endometrial receptivity [17–20]. Additionally, research has confirmed that LIF signaling may be impaired in some women with implantation failure [21]. GnRH agonist therapy may restore the endometrial secretion of implantation-related factors such as HOXA10 and LIF, which can regulate endometrial development and permit embryo implantation and decidualization [8]. Another study showed that ovarian stimulation with a GnRH agonist partially restored the expression of endometrial integrin β3 and improved uterine receptivity in mice [22].

One interesting observation in our study was that the mean level of progesterone on the day of administration in the HRT cycle was significantly higher than that in the GnRH agonist–HRT cycle (0.54 ± 0.47 vs. 0.38 ± 0.29 ng/mL; *P* = 0.000). This might have been due to a premature rise in the luteinizing hormone level in the hormonal stimulation protocol without a GnRH agonist, which reduces the “receptive window” of the endometrium [23].

The NC protocol is one of the most frequently and widely used FET protocols in ART [24]. In a large analysis, however, the GnRH agonist–HRT protocol was associated with a higher live birth rate when compared with an NC protocol for frozen–thawed blastocyst-stage transfer cycles [25]. Furthermore, another study suggested that approximately two-thirds of implantation failures are caused by impaired endometrial receptivity and asynchronized embryo–endometrial crosstalk, while embryo quality is estimated to be responsible for the remaining one-third of cases of implantation failure [26]. We also found that the live birth rate with our NC protocol was significantly lower than that in the GnRH agonist–HRT protocol, which may have been due to the “displaced window of implantation” in patients with RIF [27]. However, this conclusion needs to be confirmed.

This is the first study to evaluate the optimal approach to prepare the endometrium in FET cycles of older patients with RIF. One limitation of this study is its retrospective and single-institution design. However, through the analysis of a large sample using a multivariate logistics model, some confounding factors were eliminated. Another limitation is that the type of endometrial preparation was selected on the basis of the physician’s preference, and this might have introduced bias. Moreover, blastocyst transfer was slightly higher in the GnRH agonist–HRT group than in the other groups. Even if a Cochrane review had shown that blastocyst transfer in ART was less effective for live birth outcomes than cleavage-stage embryo transfer, this is still a bias that needs to be considered [28].

## Conclusions

This 5-year study has provided robust evidence regarding the reproductive potential of women of advanced reproductive age with RIF. The live birth rate following the GnRH agonist–HRT protocol was better than that following the other two protocols, while the miscarriage rate was not affected. Prolonged pituitary downregulation with a long-acting GnRH agonist may be involved in regulation of the endometrial genetic alterations in patients with RIF. Further multicenter randomized clinical trials are needed to confirm the possible benefits of a GnRH agonist–HRT protocol for older patients with idiopathic RIF. Additionally, the precise mechanism needs to be further investigated.

## Data Availability

Not applicable.
